# Drug repurposing for rare: progress and opportunities for the rare disease community

**DOI:** 10.3389/fmed.2024.1352803

**Published:** 2024-01-17

**Authors:** Anneliene Hechtelt Jonker, Daniel O’Connor, Maria Cavaller-Bellaubi, Christine Fetro, Maria Gogou, Peter A. C. ’T Hoen, Martin de Kort, Heather Stone, Nivedita Valentine, Anna Maria Gerdina Pasmooij

**Affiliations:** ^1^Health Technology and Services Research Department, Technical Medical Centre, University of Twente, Enschede, Netherlands; ^2^International Rare Diseases Research Consortium, Paris, France; ^3^ABPI, London, United Kingdom; ^4^EURORDIS-Rare Diseases Europe, Paris, France; ^5^Fondation Maladies Rares, Paris, France; ^6^Department of Neurology, Great Ormond Street Hospital for Children, London, United Kingdom; ^7^Department of Medical BioSciences, Radboud University Medical Center, Nijmegen, Netherlands; ^8^EATRIS ERIC, European Infrastructure for Translational Medicine, Amsterdam, Netherlands; ^9^CURE ID, Office of Medical Policy, Center for Drug Evaluation and Research, U.S. Food and Drug Administration, Silver Spring, MA, United States; ^10^Global Product Innovation, Pharmanovia, Value Added Medicines Committee, Medicines for Europe, Basildon, United Kingdom; ^11^Dutch Medicines Evaluation Board, Utrecht, Netherlands

**Keywords:** drug repurposing, rare diseases, orphan drugs, unmet need, innovation, therapy development

## Abstract

Repurposing is one of the key opportunities to address the unmet rare diseases therapeutic need. Based on cases of drug repurposing in small population conditions, and previous work in drug repurposing, we analyzed the most important lessons learned, such as the sharing of clinical observations, reaching out to regulatory scientific advice at an early stage, and public-private collaboration. In addition, current upcoming trends in the field of drug repurposing in rare diseases were analyzed, including the role these trends could play in the rare diseases’ ecosystem. Specifically, we cover the opportunities of innovation platforms, the use of real-world data, the use of artificial intelligence, regulatory initiatives in repurposing, and patient engagement throughout the repurposing project. The outcomes from these emerging activities will help progress the field of drug repurposing for the benefit of patients, public health and medicines development.

## Introduction

The last decade has seen a growing interest in drug repurposing for rare diseases (1, 2). Drug repurposing is developing an existing drug outside the scope of its original indication, with the ultimate purpose of obtaining a new regulatory-approved indication that leads to enhanced patient access opportunities, better prescribing information and treatment guidance. Drug repurposing is an innovative and attractive option for rare disease therapeutic development for several reasons. Most notably, there are currently limited options for the majority of the 6,000–8,000 rare diseases, with only 5% of diseases having an approved treatment option (3, 4). The development of new therapies for rare diseases is often challenging due to factors such as limited patient populations, disease complexity and lack of understanding of disease pathobiology, and high development costs. Therefore, drug repurposing has the potential of being time and cost-efficient, as compared to *de novo* drug development (5). This is due in part to the potential of leveraging existing knowledge about drug properties, mechanisms of action, safety profiles, and interactions with biological systems. Furthermore, drug repurposing often involves collaboration between academia, pharmaceutical companies, and patient advocacy groups. This collaborative approach may pool resources, expertise, and data, leading to more efficient and effective drug development efforts (6).

The International Rare Diseases Research Consortium (IRDiRC) is a global public/private collaborative initiative launched in 2011 by the European Commission and the US National Institutes of Health. IRDiRC has launched several initiatives to support and stimulate drug repurposing for small population needs. Most recently, it developed the IRDiRC Drug Repurposing Guidebook, which aims to help developers navigate the rare disease landscape by identifying specific tools and practices of relevance for repurposing projects (7). The Guidebook focuses on repurposing approaches, following the same successful methodology used for the IRDiRC Orphan Drug Development Guidebook. It explores incentives, regulatory tools, initiatives, and development tools for drug repurposing, helping developers ask the right questions at the right time (8). Previously, other IRDiRC endeavors have investigated key features of successful, innovative drug repurposing projects, and the corresponding business and funding models (9). As such ten factors were found to significantly contribute to the outcomes of repurposing projects. These include:

- the clear identification of unmet rare disease patients needs
- embracing innovation
- solid collaboration with patients
- gathering of information on disease prevalence, patient numbers, drug pharmacology and disease etiology
- drug industrial property status
- data gathering from inside and outside the public domain, such as off-label use
- data from past clinical studies
- the need for extended non-clinical and clinical studies
- the need for a collaborative funding framework
- early interactions with both regulatory bodies and payers.

Moreover, the IRDiRC Task Force on Data Mining and Repurposing made recommendations for investment in both research and infrastructure for drug repurposing to accelerate therapy development, which has been taken up by several funders, such as the European Commission, European Joint Programme for Rare Diseases, AFM-Telethon and Fondation Maladies Rares. These recommendations aim to improve the capture and sharing of self-reported patient data, facilitate better integration of existing research data, increase experimental testing capacity, and sharing of rare diseases research and development expertise throughout the repurposing process (10).

## Focus on practical implementation: case studies

The following cases illustrate different aspects of repurposing projects helping to contextualize the different approaches and considerations.

### Propranolol: the importance of sharing clinical observations in the public literature, use of novel regulatory route through the paediatric use marketing authorisation, seeking scientific advice

Propranolol belongs to the Beta blocking class of medicines and is a competitive antagonist at both the beta1- and beta2 adrenoceptors. Traditionally prescribed primarily in the management of cardiovascular conditions such as hypertension, the effect of propranolol on hemangiomas was discovered when treating a heart condition in a baby who also had a hemangioma. In the pivotal published case report, the clinical observation was reported that treating a heart condition with propranolol also resulted in rapid regression of a hemangioma.

A patent application was filed by Bordeaux University with the support of their tech-transfer office in 2008 (11). Pierre Fabre laboratories were contacted due to their experience in pediatric formulations, which had to be prepared for the treatment. The company obtained an exclusive license from Bordeaux University and funded a randomized, adaptive phase II/III study in infants with proliferating infantile hemangiomas (12). In 2014, propranolol received a pediatric Marketing Authorization Application from the US Food and Drug Administration (FDA), after the previous Orphan Drug Designation in 2008, and the trial showing that propranolol was effective in the treatment of infantile hemangioma (13, 14). This case is a good example of a public/private partnership showing that academic institutions and private companies can complement each other.

The study on propranolol in hemangioma ultimately led to the development of a novel oral formulation, resulting in a licensed medicine indicated in the treatment of proliferating infantile haemangioma requiring systemic therapy, authorised via a Paediatric Use Marketing Authorisation (PUMA) (15) in Europe. PUMA is a dedicated marketing authorisation covering the indication(s) and appropriate formulation(s) for medicines developed exclusively for use in the paediatric population and includes several important regulatory incentives such as periods of data and market protection. Of note the Applicant received Scientific Advice on the non-clinical and clinical aspects of the development programme.

Furthermore, based on the better understanding of propranolol’s mechanism of action including vasoconstriction, inhibition of angiogenesis and induction of apoptosis and following the success in infantile capillary haemangiomas, its potential use is being explored in other rare conditions such as Hereditary Haemorrhagic Telangiectasia, von Hippel–Lindau disease, soft tissue sarcoma, Cerebral Cavernous Malformations, and Lafora disease (16). This demonstrates how a single repurposing project, based on an initial clinical observation can successfully expand into other conditions over time.

### Fenfluramine: the rehabilitation of a withdrawn medicine in a patient group that showed a favorable benefit–risk balance

Fenfluramine is a serotonin-releasing agent acting through the release of serotonin by disrupting vesicular storage of the neurotransmitter and reversing serotonin transporter function. Its active metabolite norfenfluramine also acts as a norepinephrine-releasing agent and, at higher concentrations, as a dopamine-releasing agent.

It was launched in the 1960’s as an appetite suppressor for patients with obesity (usually combined with phentermine). Fenfluramine was withdrawn from the markets in the 90’s due to severe cardiovascular adverse events, such as heart valve problems and pulmonary hypertension. However, some clinical reports already showed a positive effect of its use on seizure management in patients with co-existing epilepsy. This evidence led a group of Belgian researchers to investigate its antiepileptic properties. Randomised clinical trials confirmed the antiseizure properties. For Dravet syndrome two pivotal studies showed a reduction in seizure frequency compared to baseline, i.e., 72.4% for fenfluramine and 17.4% for placebo, and 63.1% for fenfluramine and 1.1% for placebo, respectively, (17). Although safety data did not indicate similar cardiovascular problems in Dravet Syndrome patients exposed to 2–4 fold lower doses, the number of subjects exposed, and duration of exposure was limited. Hence, upon the approval by FDA and European Commission in 2020 of fenfluramine solution (Fintepla®) as add-on treatment for seizures associated with Dravet syndrome, the EMA requested a rigorous approach to echocardiogram monitoring and strict decision criteria for stopping the treatment (17). In 2023, fenfluramine was approved by the European Commission for another rare disease indication for seizure treatment in Lennox–Gastaut syndrome (18).

The example of fenfluramine shows that even though a drug has been withdrawn from the market based on safety concerns, it still could be considered for another indication where benefits may outweigh risks. In parallel, experimental models of rare diseases can facilitate the process of repurposing, providing biological confirmation of pharmacological properties.

### Alpelisib: benefits of working early with industry

Alpelisib, an inhibitor of PIK3CA, has been studied in patients with PIK3CA-mutated, HR-positive, HER2-negative breast cancer. PIK3CA-related overgrowth spectrum (PROS) is a group of genetic disorders caused by PIK3CA mutations and characterized by malformations and tissue overgrowth. Identical mutations in the PI3K pathway are seen in PROS and cancer (19). Canaud’s research group at Necker hospital, Paris decided to explore the therapeutic potential in PROS and requested access to the molecule. Novartis provided Canaud’s group with the molecule. After achieving positive outcomes first on PROS mouse models and then on two patients suffering from extremely severe PROS, Canaud’s group gained a temporary use authorization by the French regulatory authorities to administer alpelisib (BYL719) to 17 additional PROS patients. The study was published in 2018, supporting PIK3CA inhibition as a promising therapeutic strategy in patients with PROS (20). In 2019 Novartis received FDA approval of alpelisib (Piqray®) for metastatic breast cancer (EMA approval in 2020). The same galenic formulation (tablets) was used in PROS and cancer. In 2022, the FDA granted accelerated approval to alpelisib (Vijoice®) to treat severe manifestations of PROS (21). In addition, the application was granted breakthrough designation and orphan drug designation by the FDA. Efficacy was evaluated using real-world data from the single-arm clinical study EPIK-P1 in PROS patients who received alpelisib as part of an expanded access program for compassionate use (22). The EMA gave an Orphan Designation (EU/3/21/2420) for treatment of PROS in 2021. In 2023, however, Novartis withdrew its application for a marketing authorization of alpelisib (Vijoice®) for the treatment of PROS in the European Union (23). The withdrawal of the application was based on the time needed to acquire further data to support the benefit–risk assessment of Vijoice (24). Novartis intends to submit a new marketing authorization application once the prospective data become available.

Alpelisib is an example of drug repurposing through an early and effective collaboration between academia and industry. Ongoing additional clinical trials are conducted to further understand the long-term efficacy and safety of alpelisib in PROS.

## Trends in repurposing: initiatives to move the field

Drug repurposing is a growing trend in recent years due to lack of therapies for many rare diseases (25). Several global initiatives are ongoing to support drug repurposing. These have been summarized in Table 1, together with several examples. We will outline these trends in more detail below.

**Table 1:** Overview of trends and examples of corresponding initiatives.

Trends	Examples
Innovation platforms	Remedi4ALL (26), NCATS New Therapeutic Uses program (27), NHS England Repurposing Program (28), LifeArc toolkit (29), NeDRex.net (30)
Use of real-world data	FDA/NCATS CURE ID (31), Cure Drug Repurposing Collaboratory (32)
Use of AI in repurposing	EveryCure (33), REPO4EU (34), Biovista (35), Broad Drug Repurposing Hub (36), OpenTargets (37)
Regulatory initiatives in repurposing	STAMP (28), EMA Pilot to support academia (38), FDA Initiative to update old labels (39), Medicines Repurposing International Network (MERIT) (28)
Patient engagement	IMI Paradigm (40), IMI Prefer (41), SIMPATHIC (42)

### Innovation platforms

Different international innovation platforms have been launched to support the drug repurposing community. REMEDi4ALL (repurposing medicines for all) is an EU-funded 5-year research initiative aimed at creating a sustainable and globally connected platform and driving forward the repurposing of medicines in Europe. The REMEDi4ALL consortium brings together a unique combination of expertise to address the complexities of drug repurposing with infrastructure capacities accessible to the research community (26). Led by EATRIS, the European infrastructure for translational medicine (43–45), 24 organizations have joined forces to build a catalogue of drug repurposing services and alleviate the current systemic bottlenecks in drug repurposing.

The NHS England Medicines Repurposing program is a multi-agency initiative supported by the Department of Health and Social Care, the Medicines and Healthcare products Regulatory Agency (MHRA), the National Institute for Health and Care Excellence, the National Institute for Health and Care Research and NHS England. It aims to identify and progress opportunities to use existing medicines in new ways (28). Key objectives include identifying and developing opportunities to repurpose prioritized medicines to improve outcomes, patient experience and value for money; to support and advance innovative research into medicines that might be repurposed and adopted into the NHS; and to facilitate and encourage the licensing of repurposed medicines to support clinical decision making and improve equity of access. The first success of the Medicines Repurposing Program was reported recently (46). Anastrozole has been authorized by the MHRA in November 2023 as a preventive option in post-menopausal women at increased risk of breast cancer, including those with a significant family history of the disease. It was recommended as a preventive option by NICE in 2017, however, with the treatment being unlicensed, uptake remained low. The Medicines Repurposing Program was instrumental in getting the treatment on label including facilitating scientific advice and drug licensing. Accord Healthcare was selected through an open competitive process to undertake the licensing work on a not-for-profit basis. The Medicines Repurposing Program will work with the MHRA and the British Generic Manufacturers Association to ensure other companies that make anastrozole adopt the new licensed indication.

### Use of real-world data

Real-world data (RWD) plays a crucial role in drug repurposing by providing insights into the safety, effectiveness, and usage patterns of existing drugs in diverse patient populations. Real-world data from electronic health records, claims databases, clinician and patient reports, and other sources can be analyzed to identify potential associations between existing drugs and new therapeutic indications, as well as for post-marketing surveillance of both the safety and efficacy of approved products. CURE ID, an online platform created through a collaboration between the FDA and the National Center for Advancing Translational Sciences, part of the National Institutes of Health (NCATS/NIH) facilitates the reporting of novel uses of existing drugs for challenging infectious diseases (31). It is now being expanded to include other areas of high unmet medical need beyond infectious diseases, including rare cancers and rare genetic diseases. It allows healthcare providers, patients, and care partners the opportunity to share real-world clinical outcomes for drugs used in new conditions, populations, doses, or combinations. This crowdsourcing initiative aims to accelerate the development of treatments for rare and neglected diseases by collecting and analyzing medical information. This has been particularly important for rare diseases for which randomized clinical trials (RCT) would be difficult, if not impossible to conduct. For example, *Balamuthia mandrillaris* is an amoeba that causes meningoencephalitis in a handful of cases a year. It has a fatality rate of >90% and because of its rapid and serious nature, cannot readily be studied in an RCT. Initial case reports from clinicians and care partners in CURE ID received through contact with patient care teams has shown that a repurposed drug, nitroxoline – a quinolone antibiotic approved in Europe for UTIs for 50 years – appears to be a promising treatment for Balamuthia (47). In addition, the ability to extract data directly from electronic health records (EHRs) on drug repurposing is expanding opportunities to learn from real-world data to identify potentially safe and effective treatments for rare diseases. In collaboration with the CURE Drug Repurposing Collaboratory and other partners, CURE ID has developed the “EDGE Tool,” which enables the automated extraction of the CURE ID case report form for selected inpatient conditions. This tool is now being expanded to explore drugs that are being repurposed for rare diseases, such as unusual causes of meningitis (32, 48).

### Use of AI

Utilizing advanced computational methods, machine learning, and artificial intelligence (AI) has become increasingly common in identifying potential drug repurposing candidates. These technologies can analyze large knowledge graphs with information on biological, clinical, and chemical properties mined from literature and databases, to predict new therapeutic uses for existing drugs. An example of such a platform is the EveryCure platform, that uncovers new therapeutic uses for existing drugs by creating an open-source database of repurposing opportunities (33). It gathers and analyzes data from various sources, including PubMed, clinicaltrials.gov, and medical records, using natural language processing and machine learning algorithms. Based on collaborating with disease experts and pharmaceutical companies, these assessments should be further refined with research organizations to conduct clinical trials for the most promising opportunities.

Alongside this initiative, another EU-funded project, REPO4EU will establish a comprehensive European and global platform for mechanism-based drug repurposing, redefining diseases applying advanced bioinformatics and artificial intelligence to real-world Big Data (34). This is a disease-agnostic platform, that aims to move towards a personalized medicine approach for drug development. Other online platforms like OpenTargets (37) and Biovista (35) use AI to summarize supporting evidence from the literature and calculate association scores between diseases and drugs or drug targets.

### Regulatory initiatives

Building on the European Commission’s Expert Group on Safe and Timely Access to Medicines for Patients (STAMP) discussions on a proposal for a medicine repurposing framework, the European Medicines Agency and the Heads of Medicines Agencies (HMA) launched a pilot project in 2021 to support the repurposing of medicines (38, 49). The goal of this initiative is to assist not-for-profit organizations and academic institutions and individuals in collecting or producing enough evidence to gain formal regulatory approval for the new use of an established medicine. It is open to those with a keen interest in repurposing authorized medicines for new indications in public health and who have a valid scientific rationale, seeking scientific guidance from regulatory authorities. Since there is a growing number of publicly funded and regulator-initiated initiatives on drug repurposing in the beginning of 2023 the Medicines Repurposing International Network (MERIT) was created, consisting of 12 organizations aiming to share expertise and learnings with each other (personal communication; (28)).

The increased interest in drug repurposing is also supported by the European Commission’s recent draft proposal for the EU pharmaceutical legislation of April 2023 (50). Special provisions and incentives are included for repurposing to make it easier for researchers and not-for-profits to materialize their research into authorized medicines. The draft text of Article 48 indicates that “An entity not engaged in an economic activity (‘not-for-profit entity’) may submit to the Agency or to a competent authority of the Member State substantive non-clinical or clinical evidence for a new therapeutic indication that is expected to fulfil an unmet medical need. The Agency may, at the request of a Member State, the Commission, or on its own initiative and on the basis of all available evidence make a scientific evaluation of the benefit–risk of the use of a medicinal product with a new therapeutic indication that concerns an unmet medical need.” The draft proposal for the EU pharmaceutical legislation is still under discussion, and it remains to be seen what the final text will be, but it is encouraging to see that the value of repurposing initiatives is recognized.

### Patient engagement

Patient engagement is becoming increasingly important in drug repurposing initiatives, and patients can be the drivers of the drug repurposing process, as they can bring together the parties and funds for successful drug repurposing projects. Consequently, engaging patients throughout the entire drug repurposing process, starting from the early discovery and preclinical phase, across clinical trial design and implementation to regulatory decision making and product lifecycle, is essential to ensure that the repurposed drug meets the needs of patients (51). Patients can provide not only valuable insights into the effectiveness of drugs for different conditions but also help to identify new uses for existing drugs by recording side effects of medication. In addition, two previous IMI projects Paradigm and Prefer focused on tools for patient engagement by providing a Patient Engagement Toolbox (52) and recommendations on the conduct of patient preference studies, and how they can inform regulatory decision making (40, 41, 53). The Horizon Europe funded project SIMPATHIC, aiming for acceleration of drug repurposing for rare neurological, neurometabolic and neuromuscular disorders, will develop training modules for patient representatives to act as drivers of the drug repurposing pathway. REMEDi4ALL will develop complementary training modules for patients and patient engagement as key component for optimizing drug repurposing.

## Conclusion

The rare diseases research community has invested considerable efforts in drug repurposing to create much needed therapeutic solutions. A significant part of current orphan drugs in development are known drugs (6). An increasingly large part of the development of these repurposed drugs is the result of public-private-people partnerships, with patients, clinicians, academics, and companies working together. In this paper, we analyzed several initiatives and cases of successful drug repurposing, drawing out the lessons learned and highlighting potential opportunities. We also looked at the outcomes from emerging activities which can help progress the field of drug repurposing for the benefit of patients, public health, and medicines development.

## Data availability statement

The original contributions presented in the study are included in the article/supplementary material, further inquiries can be directed to the corresponding author.

## Author contributions

AJ: Writing – original draft, Writing – review & editing. DO’C: Writing – original draft, Writing – review & editing. MC-B: Writing – original draft, Writing – review & editing. CF: Writing – original draft, Writing – review & editing. MG: Writing – original draft, Writing – review & editing. P’T H: Writing – original draft, Writing – review & editing. MK: Writing – original draft, Writing – review & editing. HS: Writing – original draft, Writing – review & editing. NV: Writing – original draft, Writing – review & editing. AP: Writing – original draft, Writing – review & editing.
